# Impact of COVID-19 on Utilisation of Funds by People With Disabilities: Lessons Drawn From the Australian National Disability Insurance Scheme

**DOI:** 10.34172/ijhpm.2023.7663

**Published:** 2023-09-09

**Authors:** Yu Zhang, Satish Chand

**Affiliations:** School of Business, University of New South Wales, Canberra, ACT, Australia

**Keywords:** NDIS, COVID-19, People With Disabilities, Personalised Insurance, Australia, Utilisation of Funds

## Abstract

**Background:** COVID-19 pandemic has affected everyone, especially people with disabilities (PwD). While there has been qualitative research on the impact of the pandemic on PwD in Australia, little quantitative evidence has been produced on the magnitude of this impact.

**Methods:** A range of descriptive analytic methods are employed on the data merged from the National Disability Insurance Scheme (NDIS) and COVID-19 data on national, state, and regional levels to compare the expenditure of the NDIS participants who are in pandemic-affected regions and time periods with those that are not. Regression analysis is also performed to estimate the participants’ utilisation of funds using explanatory variables drawn from the NDIS, COVID-19, and lockdown policy information.

**Results:** Our analysis reveals that: (1) the pandemic reduced expenditure of the NDIS participants by approximately A$ 31.2 million, equal to 8.85% of the total expenditure over five quarters for the state of Victoria (VIC) alone; (2) the contractions in expenditure lasted for up to two quarters during the pandemic; (3) the reductions in expenditure were largely associated with the limited mobility imposed via lockdowns compared to the impaired access to services; and (4) the spread of COVID-19 that led to restrictions on mobility of people had a bearing on utilisation of funds by NDIS participants in the subsequent quarter.

**Conclusion:** COVID-19 has affected the expenditure of the PwD in Australia. We overlaid the NDIS data on the COVID-19 outbreaks to estimate the impact of the pandemic on expenditure and utilisation rate of the funds allocated to the NDIS participants. Our findings point to potential policy interventions to mitigate some of the adverse consequences of similar nationwide emergencies.

## Introduction

**Key Messages**

**Implications for policy makers**

Based on the results of our study, the following practical recommendations for policy-makers are made:

- The National Disability Insurance Scheme (NDIS) could prepare and publicise policies to underwrite access to services for people with disabilities (PwD) such that they are able to purchase necessary services during national emergencies such as COVID-19 pandemic.
- These policies need to remain in effect for at least two quarters after the start of such emergencies.
- Under national emergencies, the NDIS may prioritise assistance to some participant cohorts, such as those who had higher mobility and those spending their funding on daily necessities (such as consumables and transportation).
- PwD may be granted exemption to the restrictions placed on mobility of people during emergencies, and such exemptions may be targeted to allow access to specific services.

**Implications for the public**

The COVID-19 has severely affected people with disabilities (PwD) in Australia from accessing services funded through the National Disability Insurance Scheme (NDIS). Based on extensive analysis of available data, we show that: (*i*) funds were under-spent by NDIS participants during the pandemic; (*ii*) such under-spending was more significant among specific participants, such as those living independently or using funds on daily necessities; and (*iii*) spread of COVID-19 and mobility restrictions jointly contributed to the under-spend. Consequently, management plans that factor in the limited mobility during national disasters can ameliorate some of the difficulties faced by PwD in the community.

COVID-19 pandemic has affected everyone, and especially the people with disabilities (PwD). Access to healthcare support for PwD received widespread attention, and related research has been undertaken worldwide, including Europe,^1^ the United States,^2^ China,^3^ South Africa,^4^ Iran,^5^ England and Australia,^6^ and others.^7,8^ Within Australia, the pandemic impaired the implementation of the National Disability Insurance Scheme (NDIS) which allocates funds to eligible participants for purchase of services. During the pandemic, many providers of services withdrew from the market,^9^ making access to services for the participants difficult while restrictions placed on mobility of the public compounded this problem.^10^ These problems were exacerbated by impaired in-person services such as medical consultations,^11^ limited access to telehealth services,^12^ and insufficient knowledge of the impact of coronavirus young PwD and their families.^13^

 Learning the impact of such national emergency is important for policy-makers of the Federal Government and the NDIS. While there has been some qualitative research on the effects of the pandemic on PwD,^14^ little is known about the magnitude of its impact on PwD’s utilisation of their allocated budget.^15^ This study analyses data published by the NDIS, together with the COVID-19 data, to explore the impact of the pandemic on NDIS participants’ expenditure and utilisation of funds. To this end, a quasi-experimental approach is used to compare the expenditure of the participants who were in pandemic-affected regions and time periods with those who were not. The results are reported through a range of descriptive statistics, followed by regression analysis to decipher the role of COVID-19 and restriction policies in affecting the participants’ utilisation of funds. Our analysis assists in answering three primary research questions:

- Question 1: What was the quantum of under-expenditure by NDIS participants during the pandemic?
- Question 2: What were the main drivers for the under-utilisation of the funds?
- Question 3: How did these drivers influence the utilisation of funds by the participants?

New lessons learnt from the analysis includes the lagged effect of the pandemic on expenditure of NDIS participants, as well as the detrimental impact on the utilisation of funds from the restrictions placed on their mobility. All these findings point to potential policy interventions to mitigate some of the adverse consequences of similar nationwide emergencies.

## Background

###  Australian National Disability Insurance Scheme

 The Australian NDIS was designed to shift Australian disability services from government block-funding model to personalised insurance model, resulting in both an increase in the quantum of funds made available and greater choice and control over services accessed by participants of the NDIS.^16^ Similar personalised insurance scheme is also employed in other countries, such as the National Health Service in the United Kingdom,^17^ Social Security Disability Insurance in the United States,^18^ and Disability Insurance program in the Netherlands.^19^

 The NDIS is jointly funded by Australian Commonwealth and State and Territory Governments, with its implementation commenced in 2013.^20^ It has more than 500 000 participants covered with an annual budget of A$35.8 billion for 2022-2023.^21^ Through NDIS, eligible PwD develop plans based on their individual situation and specific needs, and receive a budget with which to purchase services and supports required to meet their needs and achieve plan goals. A wide range of disability types are covered by the scheme, including disabilities from birth as well as those due to disease, injury or accident. Participants of the scheme have two options in terms of their living arrangements, namely supported independent living (SIL) and specialist disability accommodation (SDA). Participants in SIL can choose to live independently while those in SDA usually require extensive in-house support. All participants are afforded three types of support with funding allocated for core services, capacity building, and capital investments.^22^ Since initiation, the NDIS has made significant progress in improving access to the services and support for the participants,^23^ however the implementation of the scheme was impaired by the COVID-19 pandemic.

###  COVID-19 in Australia and Responses

 The first outbreak of COVID-19 in Australia was recorded in March 2020, with cases reported in Sydney and Melbourne. By June 2020, the state of Victoria (VIC) saw the second wave where most of the cases were locally acquired. During this outbreak, infections were reported within aged care facilities and among PwD and healthcare workers.^24,25^ Starting from late June 2021, Australia saw the third wave of infections, with the source of transmission being in the state of New South Wales (NSW). The infections spread to VIC in early August and reached Queensland (QLD) by October. The number of confirmed cases climbed quickly, surpassing that from the previous outbreaks by September 2021 even though vaccinations were largely applied. Meanwhile, outbreaks within aged care facilities and among staff employed there increased in both NSW and VIC.^26^

 In response, individual State and Territory governments put in place policies and procedures to contain the spread of the infections. New regulations were introduced after the second outbreak through a revised management plan to protect PwD. In July 2020, the National Disability Services urged all disability service providers to adopt the advice of the Department of Health and Human Services on infection control, including wearing of face masks in workplaces. In August, new COVID-19 infection control procedures were developed to prevent disability workers from spreading the virus in multiple disability facilities and individual homes.^27^ Within the NDIS, service providers were encouraged to create their own plans in response to the pandemic.^6^ Consequently, most service providers cutback on their operations with the last few suspending their operations altogether at the height of the pandemic.^28^ This then led to severe shortages of some services, lengthening waiting lists for the curtailed services.

 Protocols of test, isolate, and treatment were employed extensively to contain the spread of COVID-19 virus. Additionally, intermittent lockdowns were placed, restricting the mobility of people in regions where the outbreaks were severe. For instance, VIC Government introduced unprecedented public restrictions in June 2020 including impositions of multiple periods of lockdowns limiting movement of the residents within set geography and travel times. These impositions curtailed the spread of the virus but also disrupted access to services for the PwD. Later in July and August, VIC ratcheted up the restrictions by imposing curfews, closed schools and most retail outlets, and allowed movement just for essential work and exercise.

###  Impact of COVID-19 on People With Disabilities

 Existing literature has reported the impact of COVID-19 on the PwD. The Center for Disease Control and Prevention, for example, analysed impact of COVID-19 infections and revealed that older adults, racial and ethnic minority groups, and those with underlying chronic health conditions were severely impacted compared to the rest of the population.^29^ These findings are aligned with previous research outcome which showed that people with underlying neurological conditions were at a higher risk of dying from seasonal influenza and other respiratory-based diseases.^30^ In addition, a recent analysis of national mortality data for deaths attributed to pneumonia prior to the COVID-19 outbreak indicated a 2 to 6 times higher death rate due to pneumonia among people with intellectual and developmental disabilities compared to those without these disabilities.^31^ Similarly, a comparative analysis between rural and urban areas in the United States revealed that pandemic growth in rural areas was mostly driven by outbreaks within institutional settings such as prisons, meat and poultry processing plants, and nursing homes while those in urban areas were more widespread.^32^

 Little is known on the magnitude of impact of COVID-19 on PwD in Australia. Researchers have relied on targeted surveys to collect information on the experiences of students with disabilities during COVID-19,^33^ satisfaction of the NDIS participants with the services and supports provided during the pandemic,^9^ experiences with remotely delivered NDIS consultations,^34^ COVID-19 outbreaks among PwD living in residential care facilities,^26^ and implied risk of severe health outcomes within PwD given their higher rates of chronic morbidities.^35^ However, none of the existing studies produces quantitative estimates of the impact of COVID-19 on the expenditure of PwD.

 Knowing the impact of COVID-19 pandemic is important for policy-making. The magnitude of such an impact has a direct bearing on the Federal Budget given the size of the outlay. Demonstrating the decreased spending of PwD can indicate that they were unable to purchase the services and supports they needed during the pandemic, pointing to market failure and unmet need for support among PwD. This can advise policies to reduce poverty during the pandemic and expedite recovery in the aftermath.^15^ In addition, an understanding of the factors that drove expenditure of NDIS participants can provide insights to policy-makers of the NDIS on the trade-off between broad benefits and risks of potential side effects. Therefore, this study aims to explore impact of the COVID-19 on the NDIS participants’ expenditure and utilisation of funds using data as evidence.

## Methods

**Figure 1:**
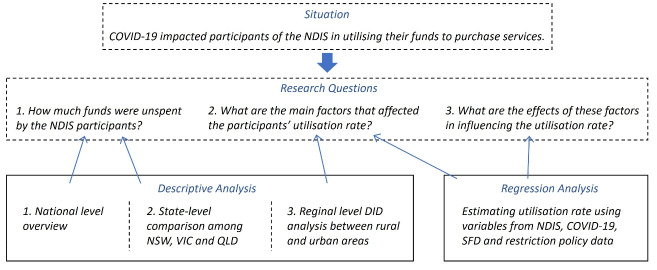
Research Framework. Abbreviations: NDIS, National Disability Insurance Scheme; VIC, Victoria; NSW, New South Wales; QLD, Queensland; DID, difference-in-differences; SFD, State Final Demand.

 We leveraged quantitative methods spanning descriptive and regression analysis to assess the impact of COVID-19 on utilisation of funds allocated to the NDIS participants. The descriptive analysis presented information from national, state-, and regional level by merging NDIS data with COVID-19 data. The regression analysis was then conducted to identify the factors that had driven participants’ utilisation of funds during the pandemic. The overall structure of the research framework is shown in Figure 1 where the situation and research questions are presented in the dashed boxes and the actions undertaken enclosed in solid lines. The specific methods are described in detail in the following sections.

###  Methods in Descriptive Analysis 

 The national level descriptive analysis aims to offer an overview of NDIS participants’ quarterly budget, expenditure, and utilisation rate across time, and present the variance of these figures. By analysing against the spread of the COVID-19 in Australia, this analysis highlighted the impact of the pandemic on the expenditure of funds from the NDIS.

 The state-level analysis compared the expenditure and utilisation rate of NDIS participants between the periods that were affected by the pandemic versus those that were not, and across regions/jurisdictions for the overlapping quarters when infection rates were very different between them. In this analysis, the states of VIC, NSW, and QLD were selected because they have the largest numbers of NDIS participants, experienced the largest numbers of COVID-19 cases, and have the largest population of all six States in Australia.^36^ Furthermore, QLD and VIC are located on the eastern seaboard flanking NSW. Residents from NSW were able to move freely across the borders (as for the rest of Australia), except for the periods when hard lockdowns were placed during the outbreaks. Based on the comparison, we calculated a counterfactual expenditure for the state that was worst affected by the pandemic compared to its neighbour States. Specifically, we created the counterfactual budget and expenditure curves for the worst impacted State (such as VIC) by averaging the quarterly budget and quarterly expenditure in the other States. Formally, we assumed the ratio between quarterly expenditure and budget in VIC is equal to that in its neighbour States. That is:


(1) $$\frac{E_{VIC}^{*}}{B_{VIC}}=\frac{E_{Ave}}{B_{Ave}}$$

(2) $$E_{Ave}=\frac{\sum {}_{i\in n}E_{i}}{n},\;B_{Ave}=\frac{\sum {}_{i\in n}B_{i}}{n}$$

where $E_{VIC}^{*}$ refers to the counterfactual quarterly expenditure in the absence of the pandemic; *B*_VIC_ denotes the actual quarterly budget in VIC; *E*_Ave_ and *B*_Ave_ are the average of the expenditure in the neighbour states and that of budget respectively; *E*_i_ and *B*_i_ refer to the quarterly expenditure and budget in state *i*respectively, and *n* denotes the total number of states. In this way, we can calculate the counterfactual level of quarterly expenditure for VIC using the following equation.


(3) $$E_{VIC}=B_{VIC}.\sum {}_{i\in n}\frac{E_{i}}{B_{i}}$$

The difference-in-differences (DID) technique was used in regional level descriptive analysis to estimate the effect of lockdowns by comparing the changes in expenditure over time between the NDIS participants of different characteristics. Specifically, we firstly partitioned the NDIS data of VIC into urban VIC and rural VIC based on the geographic matching between the local government areas and local health districts provided by the VIC Department of Health and Human Services.^37^ The purpose of this data segmentation is to allow assessment of impact of lockdowns on the level of NDIS participants’ access to services because the urban areas hold deeper markets providing healthcare services compared to the rural areas. In addition, the lockdown policies in urban VIC were more stringent compared to those in rural regions, which provides another factor for comparison. Secondly, these two segments were further divided into four by NDIS participants’ living conditions, ie, whether they are in SIL or in SDA. This segmentation assists in evaluating the impact of lockdowns on the mobility of participants, assuming that participants in SIL hold higher level of mobility than SDA participants in general, and essential healthcare services (such as access to carers) were permitted for both cohorts. Consequently, the NDIS participants in VIC were grouped into four cohorts, including Urban-SIL, Urban-SDA, Rural-SIL and Rural-SDA. A comparison between the changes in the expenditure of these four cohorts during lockdowns shed light on whether the lockdowns impacted participants’ expenditure via impairing their mobility or access to services.

###  Methods in Regression Analysis 

 Regression analysis was conducted to answer the second and third research questions. Linear regression estimates the probability of an event occurrence by fitting data to linear predictor functions which model the relationship between two or more independent variables and the one dependent variable.^38^ The linear regression algorithm was chosen because it is one of the classic and well-established machine learning techniques, especially its ability to display the statistical relationship between each of the predictors and the outcome. The general form of the linear regression model is:


(4) Y=X.β+ϵ

where *X* is the independent variable matrix in size of *m ×* (*n + 1*), assuming a dataset with *m* observations and *n* variables; *Y* is the dependent variable vector of *m × 1*; *ϵ* is the error vector of *m × 1*; and *β* represents the parameter estimate from the ordinary least squares (OLS) regression. In this paper, linear regression was used to estimate the utilisation rate of NDIS participants on the state-level information, where the explanatory variables were drawn from a cross-sectional data created by merging the NDIS, COVID-19, lockdown policy, and State Final Demand (SFD) data. The selection and explanation of variables for the regression model is presented in the Datasets and Pre-processing section. Problem of heteroscedasticity was addressed using weighted least squares method, where the OLS estimate (*β*) was updated to weighted least squares estimate ( $\hat{\beta }$ ):


(5) $$\hat{\beta }=\arg\;\underset{\beta }{\min}\sum_{i=1}^{n}\epsilon_{i}^{{*}^{2}}={\left(X^{T}.\;W.\;X\right)}^{-1}.\;X^{T}.\;W.\;Y$$

where *W* is a matrix containing the weights $w_{i}=1/\sigma_{i}^{2}$ , where $\sigma_{i}^{2}$ represents variance of the errors in the OLS regression. To obtain *σ*_i_, we performed another OLS regression using the absolute residuals to estimate a standard deviation function against the fitted values. The resulting fitted values of this regression are used as estimates of *σ*_i_.

## Datasets and Pre-processing

###  National Disability Insurance Scheme Data

 The NDIS data has been published quarterly from June 2019 on the official NDIS website.^39^ In this study, data of 9 quarters was used, that is, from the third quarter of 2019 to the third quarter of 2021. There are 103 030 observations after removing duplicate records. The attributes used for analysis contain:

- Quarter: Quarter of the data record.
- State: Australian State or Territory where the participant resides.
- Region: Service district where the participant resides.
- SIL or SDA (*SoS*): Whether the participant is in Supported Independent Living (*SIL*) or in Specialist Disability Accommodation (*SDA*). Note that the participants are either in SIL or SDA.
- Support class (*Sup*): The support classes under which the recorded participant receives funds, including Core (*Cor*), Capacity Building (*Cab*), and Capital (*Cap*) support.
- Age band (*Ag*): The range of age where the recorded participant is at. The original 9 age bands were collated into three categories: *Ag* ≤ 18, *Ag* (19-54], and *Ag* > 54.
- Participant count (*Pc*): number of participants in the service district.
- Budget (*Bu*): Amount of funds in dollars approved for the participant’s plan in the recorded quarter.
- Utilisation rate (*Ur*): Percentage of funds that the participant has used in the recorded quarter.

The first six attributes contain categorical values whereas the last three are numerical. Expenditure of NDIS participants (*Exp*) was generated by multiplying the *Bu* and *Ur*.

###  COVID-19 Entry Data and Policy-Induced Restriction Data

 To assess the impact of the COVID-19 on expenditure of the NDIS participants, the NDIS data was merged with COVID-19 related data. This includes (1) COVID-19 entry data that was aggregated by Covid19data.com.au and downloaded via a GitHub repository,^40^ and (2) policy-induced restriction data (referred to as policy data hereafter).

 As for the COVID-19 entry data, we collated two explanatory variables, namely number of daily confirmed cases (*CC*) and cases in hospital (*CH*) in quarters to align with the quarterly format of the NDIS data.

 In addition, data on the restrictiveness of policies was collected from two sources, namely restriction policies released by the NSW, VIC, and QLD government websites and the Oxford COVID-19 Government Response Tracker (OxCGRT) via a GitHub repository.^41^ These restrictions reflect the stringency of the pandemic-induced regulations, which were later considered as factors that drove the utilisation of funds by NDIS participants.

 To collate explanatory variables from the policy data of state government websites, we manually reviewed all the published policy updates and retrieved the specific content in every update regarding two key restrictions during the pandemic:

- Indoor gathering limit (*L*_in_): The maximum number of individuals who were allowed to gather indoor.
- Outdoor gathering limit (*L*_out_): The maximum number of individuals who were allowed to gather outdoor.

We converted the numerical values specified in the restrictions into categorical variables based on pre-defined criteria. Specifically, we firstly defined an average restriction value for quarterly restriction based on the following equation:


(6) $$Rst_{q}=\frac{\sum_{i=1}^{n_{q}}Rst_{i}.\;D_{i}}{90}$$

where *Rst*_q_ refers to the average restriction in quarter *q*, *Rst*_i_ denotes the restriction in period *i*, *D*_i_ is the number of days in the period *i*, and *n*_q_ means the number of time periods in quarter *q*. For instance, assume Q1 restricted indoor gatherings to a maximum of 2 people per day for 50 days, 5 people for 30 days, and 10 for 10 days, then the average restriction value for Q1, *Rst*_Q1_ = (50 × 2 + 30 × 5 + 10 × 10)/90 = 3.89 visitors per day. Subsequently, we defined criteria to convert the *Rst*_q_ into categorical format:

- For indoor gathering limit, we defined three categories, namely, maximum 10 people can gather and “no gathering is allowed” (*L*_in_ ≤ 10); limits from 10 to 20 people (*L*_in_ (10, 20]); and limits above 20 people, including “no limit for indoor gatherings” (*L*_in_ > 20).
- For outdoor gathering limit, we defined three categories, namely, maximum 20 people can gather and “no gathering is allowed” (*L*_out_ ≤ 20); limits from 20 to 100 people (*L*_out_ (20, 100]); and limits above 100 people including “no limit for outdoor gatherings” (*L*_out_ > 100).

Furthermore, we used a stringency index from the OxCGRT as another variable for restriction:

- Restrictions on internal movement (*L*_mov_): Record restrictions on internal movement between cities/regions. The index *L*_mov_ is a categorical variable where three categories are recorded, namely Class 1, 2, and 3, representing different level of restriction stringency, where Class 3 is the most stringent.

Note that the reason for selecting only *L*_mov_ from the OxCGRT is due to the focus of this study and to reduce bias by human factor. Specifically, since this study aims to explore the impact of restriction policies on the spending of the NDIS participants, those OxCGRT indices that are not related to restrictions are excluded. In addition, the OxCGRT collated restriction policies across a wide range of countries, hence the same spectrum of restrictions was applied to all the countries even though some restrictions were not imposed in Australia, such as the OxCGRT index “C5M_Close public transport.” As a result, some indices were deduced by human interpretation using the same policy. For instance, the “C1M_School closing” and “C2M_Workplace closing” indices were often deduced from the same policy which did not specify whether schools or workplaces must be shut down. We also found high correlation between *L*_in_ and “C3M_Cancel public events,” and between *L*_out_ and “C4M_Restrictions on gatherings.” Therefore, this study only used restriction indices including *L*_in_, *L*_out_, and *L*_mov_ which were all specifically stated in the polices.

###  Merged Data and Pre-Processing

 The COVID-19 entry data and policy data were merged to the NDIS data using time (Quarter) and location (State and Territory) as key attributes. In addition, the quarterly Australian SFD information was also integrated. It indicates the total value of goods and services that are traded in a state by end-consumers for consumption or investment. It was included as an explanatory variable (*SFD*) to test whether the state-level economic activity had an influence on the utilisation rate of the NDIS participants. While the budget funded to the NDIS is a small part of SFD, the Pearson correlation coefficient between *Ur* and *SFD* is low (coefficient of -0.002) thus reducing the possibility of reverse causation.

 In the descriptive analysis, the NDIS participants’ budget, utilisation rate and expenditure on the total 103 030 observations in three jurisdiction levels and across time were presented. This allows for segmentation of the data into subgroups for comparisons.

 In addition, the regression analysis used 11 attributes (*SoS, Ag, Sup, Pc, CC, CH, L*_in_, *L*_out_, *L*_mov_, and SFD) as explanatory variables to estimate the participants’ utilisation rate (*Ur*). Four steps of pre-processing were conducted to prepare for the analysis:

1. Aggregation: The overall data was aggregated by state, quarter, and the categorical NDIS variables (*SoS, Ag, *and* Sup*). The *Pc* was averaged, the *CC* and *CH* were summarized during the aggregation, and the remaining variables were joined. Meanwhile the three categorical variables were dummy coded. A total of 315 observations were generated after aggregation.
2. Normalisation: The *Pc* in each observation was divided by the state-level total participant count, making it *Pc*_r_. The *CC* and *CH* were converted to relative perspective by diving the state-level population, making them *CC*_r_ and *CH*_r_.
3. Transformation: The response variable (*Ur*) was logarithm transformed into *log*(*Ur*), meanwhile quantile transformation was performed on *Pc*_r_, *CC*_r_,* and CH*_r_, making them *Q*(*Pcr*), *Q*(*CCr*), and *Q*(*CHr*).
4. Correlation and collinearity tests: Pearson correlation and variance inflation factor measures were employed on the explanatory variables, and the results of the tests are listed in Table S1 at Supplementary file 1.

## Results of Descriptive Analysis

###  National Level

**Figure 2:**
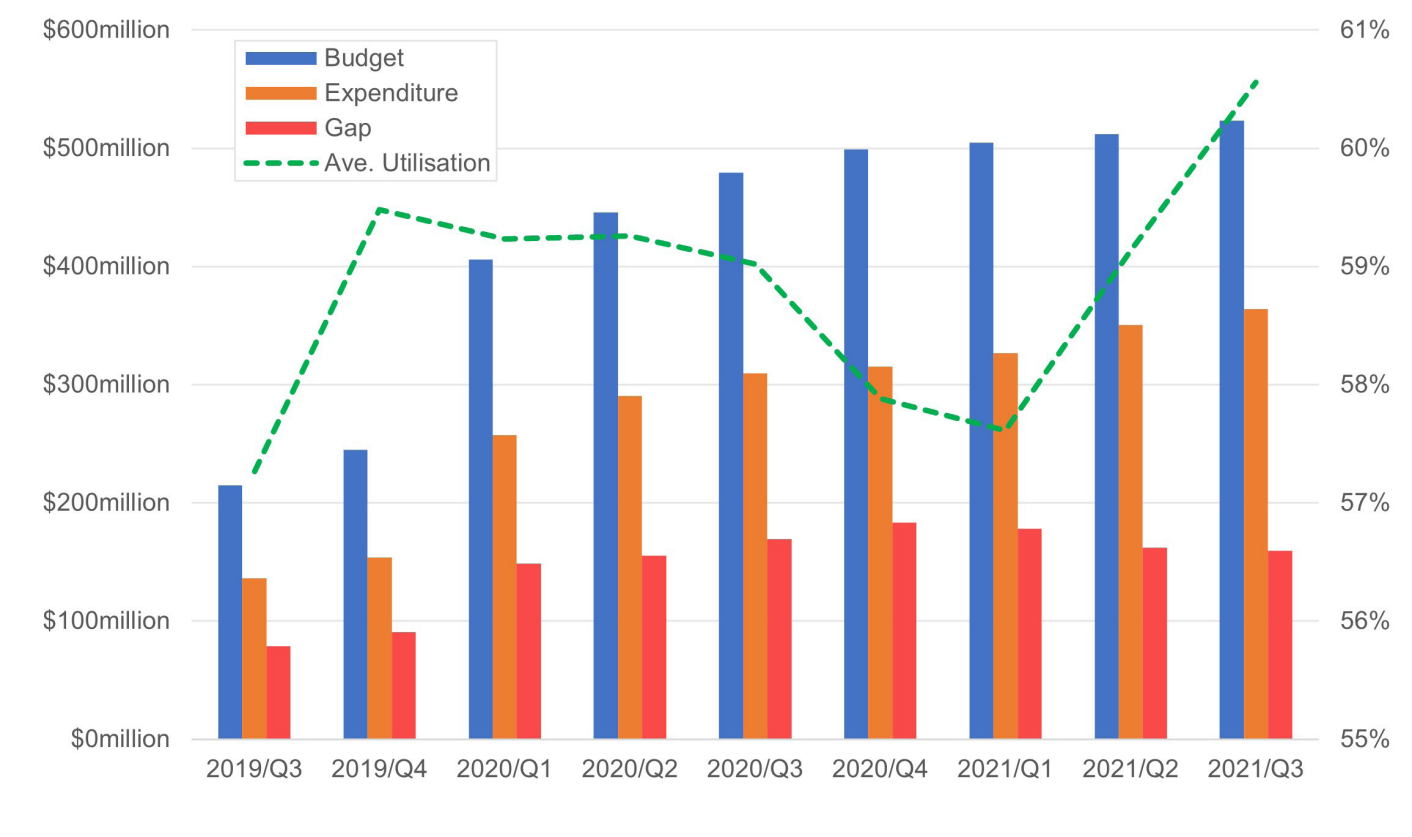
National Level Quarterly Budget, Expenditure and Gap as Shown by the Bar Chart, and Average Utilisation Rate as Shown by the Line Chart.

Figure 2 shows the quarterly budget, expenditure and gap (in dollars) of the NDIS, as well as the average (Ave) utilisation rate (in percent). The gap here refers to the margin between the budget and expenditure.

 The overall budget allocated to the NDIS participants has increased over time. Similarly, the levels of expenditure by the participants also increased over the 9 quarters. The average utilisation rate increased from 2019/Q3 to the next quarter, held up for the subsequent three quarters, dipped significantly from 2020/Q3 to the nadir of 57% in 2021/Q1 before rebounding over the next quarter. In addition, average utilisation rate is above 59% in Q1, Q2, and Q3 before dropping to a low of 55% in Q1 of 2021. Recall that Australia was affected by the COVID-19 pandemic in Q1, Q2, and Q3 of 2020, and then spent the subsequent three quarters with minimal number of cases detected, followed by another wave from the end of Q2 of 2021. The episodes of the outbreaks are broadly aligned with the variation in the utilisation rate, which suggests that the pandemic may have been responsible for the changes in utilisation rate. However, it also suggests that the pandemic may have caused a carryover effect over the utilisation rate. Factors other than the pandemic may have been in play thus the DID method is used to create counterfactual levels of expenditure in the absence of the COVID-19.

###  State Level

**Figure 3:**
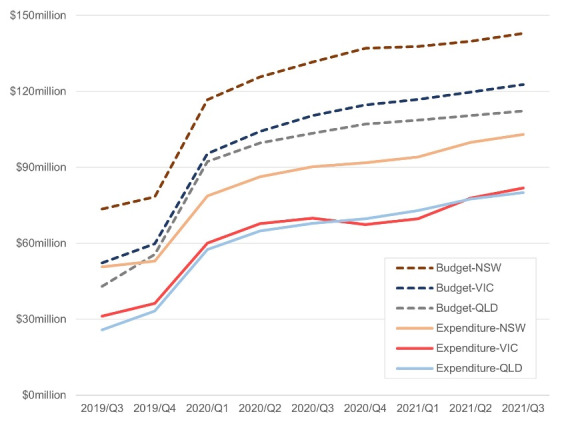
Comparison Between the Quarterly Budget and Expenditure in New South Wales, Queensland, and Victoria. Abbreviations: VIC, Victoria; NSW, New South Wales; QLD, Queensland.

Figure 3 illustrates that the budget of NDIS increased over all quarters in all three states. However, an abnormal drop is noticeable in the expenditure for VIC from the third quarter to the last quarter of 2020. This dip is noticeably absent in NSW and QLD; rather the shapes of their curves are close to being parallel suggesting that utilisation rates are similar across the three jurisdictions. We read the dip in Expenditure for VIC as being the impact of the pandemic. Recall that VIC was affected the most compared to the other states in Q1, Q2 and Q3 of 2020. Especially in 2020/Q3, VIC was responsible for most of the COVID-19 cases in Australia yet there were not many cases in the other States. By comparing the expenditure curves between NSW, QLD, and VIC, we deduce that the pandemic slowed down the rise in spending funds across the nation, indicating the carryover effect of the COVID-19 noted earlier.

 While the state-level expenditure of VIC had barely grown from 2020/Q2 ($67.78 million) to 2021/Q1 ($69.75 million), the corresponding increases for NSW and QLD from $86.35 million to $94.15 million and $64.84 million to $72.95 million, respectively, were significant. Therefore, we use the equation (3) for VIC and found that a margin of $31.2 million between the counterfactual expenditure and the actual expenditure from 2020/Q2 to 2021/Q2 for VIC. In other words, COVID-19 pandemic lowered expenditure for VIC by $31.2 million which is around 8.85% of the total expenditure and 5.51% of the total budget in these quarters.

###  Regional Level

**Figure 4:**
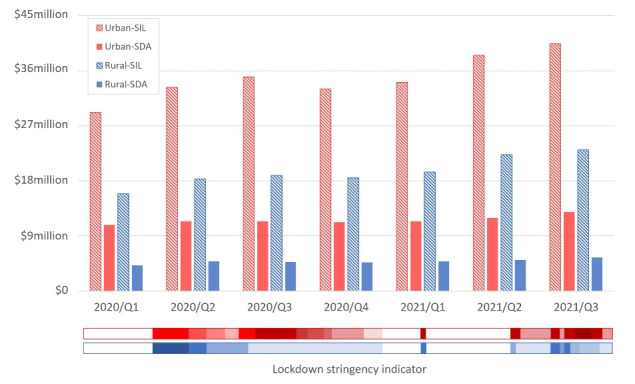
Quarterly Expenditure of the Four Participant Cohorts Grouped by Urban/Rural and SIL/SDA Associated With Lockdown Stringency. Abbreviations: SIL, supported independent living; SDA, specialist disability accommodation.

Figure 4 shows the expenditure of the four participants cohorts in VIC during the COVID-19 lockdowns, where the vertical axis measures the sum of expenditure within each cohort. A colour-coded indicator was developed based on the Timeline of Every VIC Lockdown (Dates & Restrictions)^42^ to associate the quarterly expenditure with the lockdown stringency in urban and rural VIC. Although there were six lockdown episodes imposed in VIC to the end of 2021, only five could be aligned to the available NDIS data due to the limited data availability. Depth of colour denotes the intensity of the restrictions during the lockdowns: darker colour indicates more stringent lockdown restrictions, and lighter colour means less intense restrictions. The intensity is presented based on the semantic description of the lockdown restrictions and the rules included in the restrictions. For instance, Stage 4 lockdown is more stringent than Stage 3; restriction on movement of a maximum 5 km from home is stricter than that of 25 km; and a lockdown with curfews in place is more stringent than otherwise. No colour means there was no restriction.

 Observations can be drawn by associating the lockdown timeline with the quarterly expenditure in Figure 4. Specifically, the intensity of the lockdowns had a bearing on the levels of, and variations in, the expenditure, particularly in terms of the margin of decrease after a strict lockdown. This finding is reflected by comparing between urban and rural VIC: lockdowns in urban and rural areas started at Stage 3 intensity from the beginning of Q2 of 2020 but restrictions in rural regions were gradually relaxed from Q3 while urban VIC was placed under Stage 4 (ie, more stringent) restrictions. Consequently, urban areas that had gone through stringent lockdowns witnessed larger falls in expenditure and utilisation rate compared to rural regions.

 In addition, it is noticeable that the SIL cohorts (Urban-SIL and Rural-SIL) show much larger variance than the other two cohorts in SDA. This means that the pandemic affected expenditure of the SIL (ie, the higher mobility group) participants more than their SDA-counterparts. In addition, recall that urban VIC went through more stringent restrictions compared to rural areas, yet the expenditure of Urban-SDA does not show strong fluctuations across quarters. Note that the Urban-SDA cohort experienced the same lockdown restrictions as the Urban-SIL. If the lockdowns weighed down the expenditure of participants in urban VIC to the same degree via impairing the access to services and participants’ mobility, the cohort of Urban-SDA should have shown a similar variance to the Urban-SIL. This difference leads to the proposition that the decrease in NDIS participants’ expenditure was largely associated with the crimped mobility of people compared to the impaired access to services.

## Results of Regression Analysis

 Regression analysis was performed on the merged data after pre-processing. In addition, we regressed the lagged response variable (by one quarter) using the same explanatory variables since a potential carryover effect was demonstrated in the descriptive analysis. Note that only the COVID-19 data and policy data were lagged by one quarter to estimate the utilisation rate of next quarter.

 The coefficients and related statistics of both regression results are reported in Table. The variables are grouped by the datasets from where they were collected. For instance, variables from 1 to 3 are from the policy data; variable 4 and 5 are from the COVID-19 data; and variables 6 to 9 belong to the COVID-19 data.

**Table:** The Coefficients and Related Statistic Report of the Variables in Regressing the Average Utilisation Unlagged and Lagged by One Quarter

**ID**	**Variables**	**Coefficient**	**Standard Error**	**t-Statistic**	***P *** **Value**
			**Unlagged Results**		
1	*L*_in_ ≤ 10	-0.1280	0.118	-1.083	.280
2	*L*_out_ ≤ 20	-0.0534	0.117	-0.457	.648
3	*L*_mov_ = Class3	0.0189	0.056	0.334	.739
4	*Q*(*CC*_r_)	0.0507	0.011	4.491	.000
5	*Q*(*CH*_r_)	-0.0081	0.007	-1.097	.273
6	*Sup = Cor*	1.0928	0.053	20.722	.000
7	*SoS = SIL*	0.6726	0.084	7.992	.000
8	*Ag* ≤ 18	0.0856	0.089	0.965	.335
9	*Q*(*Pc*_r_)	0.2082	0.034	6.111	.000
10	*SFD*	-0.0367	0.017	-2.098	.037
	(intercept)	-0.6216	0.059	-10.511	.000
			**Lagged Results (By One Quarter)**		
1	*L* ^*^_in_ ≤ 10	0.2829	0.114	2.475	.014
2	*L* ^*^_out_ ≤ 20	-0.2273	0.056	-4.074	.000
3	*L* ^*^_mov_ = Class3	-0.1503	0.053	-2.832	.005
4	*Q*(CC^*^_r_)	0.2126	0.042	5.052	.000
5	*Q*(CH^*^_r_)	-0.0317	0.010	-3.132	.002
6	*Sup = Cor*	1.4967	0.029	51.562	.000
7	*SoS = SIL*	0.7235	0.089	8.108	.000
8	*Ag* ≤ 18	0.0718	0.091	0.786	.433
9	*Q*(*Pc*_r_)	0.1882	0.034	5.462	.000
10	*SFD*	0.0119	0.014	0.847	.398
	(intercept)	-0.6280	0.064	-9.808	.000

Note: The * on the variables in the second result means that they were lagged response of *Ur*. The *R*^2^ for the regression model on unlagged response is 0.742, and that on lagged response is 0.942. The *P* value of Breusch-Pagan test^43^ for the regression model on lagged response is 0.2783, rejecting the heteroscedasticity hypothesis. However, the test on unlagged response is 0.0181, indicating the existence of heteroscedasticity. The endogeneity^44^ is not expected in this regression because the selected variables are either COVID-19 statistics and restriction policies, or admin information of the NDIS participants, all of which are expected to be exogenous.

 We discuss our findings from the regression analysis next:

- The lagged model fits the data better, suggesting that the effects of COVID-19 extended for a quarter. Besides, the Breusch–Pagan test indicates that the second model is appropriate. In addition, the *P* values of the restriction variables in the regression on lagged response are all statistically significant at 0.05 level, whereas those on the unlagged response are not significant. These statistics point to a one-quarter carryover effect of the pandemic and restrictions on the NDIS participants’ utilisation of their budget.
- Focusing on the regressing results on the lagged response, we find that the stringent indoor restriction during lockdowns has a negative impact on the participants’ utilisation rate in the subsequent quarter. In contrast, the strict outdoor and interstate travel polices show a positive impact. This finding suggests that when visiting homes is restricted, the participants tend to use less of their budget in the subsequent quarter. Regarding the outdoor activity and interstate travel restrictions, the participants are estimated to plan for higher expenditure if they have more freedom in the next quarter for outdoor gathering and traveling. In addition, the quarterly confirmed cases is estimated to have a positive impact on the next quarter utilisation rate, whereas the hospitalised cases shows a negative impact, with a relatively smaller coefficient indicating its weak impact on the utilisation rate.
- Among the variables from NDIS data, the variables of core services (support for daily living activities) used by participants and independently living (SIL) condition have shown strong positive impact to the utilisation rate. This suggests that the participants with higher mobility and spending their budget on daily necessities (such as consumables and transportation) increased their utilisation during the pandemic. In contrast, those living in specialist accommodation and relying on the budget for higher-cost assistance (such as home modification) or capacity building (such as finding a job) were prone to reduce their utilisation in this period. This corroborates the claim that mobility of the participants matters for their utilisation. Only the young age is not statistically significant, which may be the result of data aggregation distributing the participants in different age bands evenly across states and quarters. Finally, the SFD is not statistically significant suggesting that the broad measure of demand in the jurisdiction had negligible impact on the NDIS participants utilisation of budget.

## Discussion

 The findings of our descriptive and regression analysis demonstrated the magnitude of the impact of COVID-19 on the utilisation of funds by NDIS participants. A drop in NDIS participants’ expenditure during the pandemic was revealed using data of VIC, and it was evident that the drop was largely associated with limited mobility of people imposed by lockdowns compared to impaired access to services. By analysing the influencing factors, our regression analysis also highlighted that the spread of COVID-19 and policy-induced restrictions had placed joint impact on participants’ utilisation of funds lagged by one quarter.

 It is not surprising that expenditure of the NDIS participants was affected by the COVID-19. Impact of the pandemic to PwD, such as lacking food or basic healthcare services,^45,46^ has been well-documented.^15^ This situation may have been prevented since the NDIS budget allocated to the participants increased during the pandemic. The new knowledge contributed to the literature lies in the magnitude of the drop in the expenditure of NDIS participants due to the pandemic. Approximately A$31.2 million were unspent by the participants residing in the state of VIC, which was about 8.85% of the state level total expenditure in five quarters, and the contraction lasted for up to two quarters. These measures provide the NDIS policy-makers with a guidance for budge planning in preparation for nation-wide emergencies of a similar nature in the future. In particular, the carryover effect indicates an estimate for how long it would take for PwD to return to the pre-pandemic levels of spending after a restrictive lockdown policy.

 Another lesson learnt is that the lockdowns in VIC had a bearing on the NDIS participants’ expenditure by crimping their mobility. This result is important for NDIS policy-makers as it points to the need to allow disability assistance in moving to the source of a service, and particularly so when the service itself cannot be brought to the consumer. The case of specialist medical services is a case in point: while such services may be delivered inside a specialist care accommodation, the same may not be possible for individuals living independently. Consequently, the latter group would require assistance with transportation during a lockdown. This aligns with the results of our regression analysis where participants living independently (SIL) and using their budget for daily consumables and transportation (Core support) are more sensitive in adjusting their utilization of budget during the pandemic. This is valuable for policy-makers to consider exclusive policies for PwD, particularly those who have travelling and self-supporting (including family support) capacity, when stringent lockdown is necessary to deal with national or state-level crisis. Without this special consideration, the risk of deteriorating health conditions for PwD, particularly those relying on regular consumables, therapies, and movement, will increase.^47^

 Furthermore, our regression analysis revealed the statistical significance and impact of the broad policy-induced restrictions on the utilisation rate of the NDIS participants lagged by one quarter. Stringent lockdowns, together with the prevailing COVID-19 situation (such as increasing number of infected and hospitalised cases), had a strong impact on the lagged response of utilisation rate. Overall, the NDIS participants spent more of their budget when facing stringent indoor restrictions. This aligns with the findings of existing literature that PwD needs extra daily goods, medication, and increased expenditure associated with COVID-19 (such as antiseptic products, face masks and rapid tests) when they can only rely on limited visits during the pandemic.^45,48^ New knowledge discovered was that the utilisation rate in the subsequent quarter was negatively impacted by stringent restrictions on outdoor gathering and interstate travelling. This result provides support to the “saving-up” hypothesis which posit that PwD would plan to spend less of their allocated budget on unnecessary purchases when anticipating tough times to come.^49,50^

## Conclusion

 There is consensus in the literature that COVID-19 has adversely affected PwD in a range of aspects. The data published by the NDIS provides the opportunity to explore the impact of the pandemic on the scheme participants’ utilisation of funds. We overlay the NDIS data on episodes of the COVID-19 outbreaks to quantify the impact of the outbreaks on expenditure and utilisation rate of the funds allocated to the NDIS participants during the pandemic.

 The quarterly scheme of NDIS data was aligned with the COVID-19 outbreaks, including the periods when lockdowns were imposed. A DID method was employed to calculate the impact of the pandemic on the levels of spending of the NDIS participants. Differences between jurisdictions within Australia that were severely affected by the outbreaks compared to those who were not, that across regions within VIC, and over time were compared to create the counterfactual levels of expenditure in the absence of COVID-19. These estimates were based on available data on utilisation of the funds allocated to the individual participants — 103 030 in total records. An initial estimate revealed that the pandemic was accompanied by a drop in expenditure of approximately 9%, relative to what would have been expected in the absence of COVID-19, and such drop in expenditure lasted up to two quarters after each outbreak. In addition, we investigated the potential causes for the drop in expenditure and found that the limited mobility imposed through policies was a key reason. A follow-up regression analysis confirmed the impact of COVID-19 and policy-induced restrictions on the budget utilisation rate of the NDIS participants lagged by one quarter.

 These findings are of relevance to contemporary policy-making. First, PwD have been adversely affected by the COVID-19 pandemic — a result that is of little surprise, but the magnitude of this impact as presented here is new to the literature. Second, restricting mobility can be as harmful as the pandemic itself in terms of access to services by PwD. Consequently, management plans that factor in the limited mobility of PwD during disasters can ameliorate some of the difficulties faced by a vulnerable group in the community.

 Finally, while we have taken utmost care in estimating the impact of COVID-19 pandemic on expenditure by NDIS participants, our analysis is far from being definitive. The quantitative estimates of ours using large data sets can be complemented with detailed and individual level surveys of NDIS participants to gain their perspective on the extent to which COVID-19 affected them. Therefore, a follow-up qualitative study would be beneficial by comparing expenditure of the participants and other cohorts, meanwhile investigating their experiences on the impact of the lockdowns on their expenditure plans. Future qualitative research can also compare the experiences of NDIS participants who live dependently and independently, have gone through tight and loose lockdown policies.

## Ethical issues

 The authors declare that the data used in this research is secondary data and thus not involving any ethical issues.

## Competing interests

 Authors declare that they have no competing interests.

## Supplementary files

Supplementary file 1 contains Table S1.
