# Supplementary material for: Impact of COVID-19 on Utilisation of Funds by People With Disabilities: Lessons Drawn From the Australian National Disability Insurance Scheme
Source: Int J Health Policy Manag. 2023 Sep 9;12:7663. doi: 10.34172/ijhpm.2023.7663 (PMC10590233; doi:10.34172/ijhpm.2023.7663)
Supplement: Supplementary file 1 — contains Table S1. [file ijhpm-12-7663-s001.pdf]

Article title: Impact of COVID-19 on Utilisation of Funds by People With Disabilities: Lessons Drawn From the Australian National Disability Insurance Scheme

Journal name: International Journal of Health Policy and Management (IJHPM)

Authors' information: Yu Zhang*, Satish Chand

School of Business, University of New South Wales, Canberra, ACT, Australia

***Correspondence to**: Yu Zhang, Email: m.yuzhang@unsw.edu.au

Citation: Zhang Y, Chand S. Impact of COVID-19 on utilisation of funds by people with disabilities: lessons drawn from the Australian National Disability Insurance Scheme. Int J Health Policy Manag. 2023;12:7663. doi:10.34172/ijhpm.2023.7663

Supplementary file 1

Table S1. The Pearson correlation and VIF report of the variables in regressing the average utilisation unlagged and lagged by one quarter.

	**Pearson Correlation**										**VIF**
	**Unlagged results**										
	L_{in}	L_{out}	L_{mov}	$Q(CC)$	$Q(CH)$	Sup	SoS	Ag	$Q(Pc)$	SFD	
L_{in}	1	0.58	0.18	0.09	0.40	0	0	0	0.02	-0.43	2.21
L_{out}	0.58	1	0.03	0.18	0.21	0	0	0	-0.02	-0.54	2.40
L_{mov}	0.18	0.03	1	0.10	0.39	0	0	0	-0.02	-0.11	2.01
$Q(CC_r)$	0.09	0.18	0.10	1	0.16	0	0	0	0.06	-0.36	1.40
$Q(CH_r)$	0.40	0.21	0.39	0.16	1	0	0	0	-0.02	-0.24	1.37
Sup	0	0	0	0	0	1	0	0	0.30	0	1.45
SoS	0	0	0	0	0	0	1	-0.41	0.30	0	1.70
Ag	0	0	0	0	0	0	-0.41	1	0.18	0	1.35
$Q(Pc_r)$	0.02	-0.02	-0.02	0.06	-0.02	0.30	0.30	0.18	1	-0.01	1.27
SFD	-0.43	-0.54	-0.11	-0.36	-0.24	0	0	0	-0.01	1	1.47
	**Lagged results (by one quarter)**										
	L^*_{in}	L^*_{out}	L^*_{mov}	$Q(CC^*)$	$Q(CH^*)$	Sup	SoS	Ag	$Q(Pc)$	SFD	
L^*_{in}	1	0.42	0.27	0.46	0.42	0	0	0	0.01	0.25	2.85
L^*_{out}	0.42	1	-0.14	-0.07	0	0	0	0	0.03	0.50	3.96
L^*_{mov}	0.27	-0.14	1	0.24	0.41	0	0	0	-0.02	0.31	1.99
$Q(CC^*_r)$	0.46	-0.07	0.24	1	0.22	0	0	0	0.06	0.16	1.66
$Q(CH^*_r)$	0.42	0	0.41	0.22	1	0	0	0	0.04	0.26	1.51
Sup	0	0	0	0	0	1	0	0	0.30	0	1.50
SoS	0	0	0	0	0	0	1	-0.41	0.30	0	1.86
Ag	0	0	0	0	0	0	-0.41	1	0.18	0	1.43
$Q(Pc_r)$	0.01	0.03	-0.02	0.06	0.04	0.30	0.30	0.18	1	-0.01	1.28
SFD	0.25	0.50	0.31	0.16	0.26	0	0	0	-0.01	1	1.67

There were only none to weak correlations found between the variables from the Pearson correlation tests, and the VIF measured for each variable was smaller than 5. Therefore, these varibles were used to estimate the utilisation rate of NDIS participants.
